# Associations between quantitative ocular surface features and CT morphology of the levator palpebrae superioris complex in patients with thyroid eye disease and upper eyelid retraction: a cross-sectional study

**DOI:** 10.3389/fmed.2026.1912490

**Published:** 2026-09-29

**Authors:** Yong Jiang, Liuhong Tian, Yaohua Wang, Zhenbin Qian, Mengdi Wang, Wei Fang, Yanan Luan

**Affiliations:** 1 The Second People's Hospital of Chun'an County, Chun'an County, China; 2 National Clinical Research Center for Ocular Diseases, Eye Hospital, Wenzhou Medical University, Wenzhou, China

**Keywords:** artificial intelligence, computed tomography, levator palpebrae superioris complex, meibomian gland, tear meniscus height, thyroid eye disease, upper eyelid retraction

## Abstract

**Background:**

This study aimed to investigate the associations between artificial intelligence (AI)-quantified ocular surface parameters and computed tomography (CT)-based morphology of the levator palpebrae superioris complex (LPSC) in patients with Thyroid eye disease (TED) and upper eyelid retraction.

**Methods:**

This retrospective cross-sectional study included 67 patients with predominantly isolated TED-related upper eyelid retraction (89 eyes) between May 2023 and October 2024. AI models were used to quantify meibomian gland vagueness, meibomian gland density, and tear meniscus height (TMH). LPSC thickness and area were measured on orbital CT images. Correlation analyses, partial correlation analyses, generalized estimating equations (GEE), and exploratory parallel mediation analyses were performed.

**Results:**

Meibomian gland vagueness was negatively correlated with anterior LPSC area (*r* = −0.333, *p* = 0.002), middle LPSC thickness (*r* = −0.287, *p* = 0.008), and middle LPSC area (*r* = −0.352, *p* = 0.001). After adjustment for clinical activity score, proptosis, and upper lid marginal reflex distance (MRD1), these correlations remained significant for anterior LPSC area (*r* = −0.372, *p* < 0.001), middle LPSC thickness (*r* = −0.297, *p* = 0.009), and middle LPSC area (*r* = −0.369, *p* < 0.001). GEE analysis identified anterior LPSC area (*β* = −0.168, 95% CI: −0.291 to −0.045, *p* = 0.008) and middle LPSC area (*β* = −0.144, 95% CI: −0.236 to −0.053, *p* = 0.002) as independent factors associated with meibomian gland vagueness. TMH was positively correlated with middle LPSC thickness (*r_s_* = 0.254, *p* = 0.017) and middle LPSC area (*r_s_* = 0.348, *p* < 0.001) in bivariate analyses, but these associations were no longer significant after adjustment. Proptosis (*β* = 0.016, 95% CI: 0.009 to 0.024, *p* < 0.001) and MRD1 (*β* = −0.025, 95% CI: −0.040 to −0.010, *p* = 0.001) were independently associated with TMH. Exploratory mediation analysis showed significant indirect effects through proptosis for the associations of middle LPSC thickness with TMH and middle LPSC area with TMH.

**Conclusion:**

In this selected cohort of patients, LPSC enlargement was associated with reduced meibomian gland vagueness. The relationship between LPSC enlargement and increased TMH appeared to be indirect and may involve proptosis.

## Introduction

Thyroid eye disease (TED) is an autoimmune inflammatory disorder associated with thyroid disease and is the most common orbital disease in adults. Ocular surface disease occurs in 65.2% to 89.2% of patients with TED, with dry eye being the most frequent subtype (1–3) TED-related dry eye frequently has a prominent evaporative component; however, the ocular surface phenotype is not static and may vary with disease activity and severity (4). Its pathogenesis involves multiple factors, including increased ocular surface exposure, abnormal eyelid dynamics, structural damage to the meibomian glands, and ocular surface inflammation (1, 5–7) During the active inflammatory phase, tissue edema and ocular surface inflammation may coexist with compensatory aqueous tear secretion, whereas chronic or inactive disease may be characterized by persistent exposure, altered blinking, and more established meibomian gland structural loss (5, 6).

Morphological abnormalities and dysfunction of the meibomian glands play an important role in the development of TED-related dry eye (8–10) Patients with TED have significantly higher meibomian gland scores (Arita meiboscore) than healthy individuals, and the upper eyelid glands are more severely affected than the lower eyelid glands (11, 12). Park et al. reported that the clinical activity score (CAS), an inflammatory activity index in TED, was independently and positively associated with meibomian gland loss (13). Aghaei et al. further demonstrated that patients with moderate-to-severe TED had significantly higher upper eyelid meibomian gland scores and meibum secretion scores than those with mild TED, and that meibomian gland loss was negatively associated with tear film lipid layer thickness (12). In addition to gland density or dropout, meibomian gland vagueness has been introduced as an image-contrast parameter derived from infrared meibography. It quantifies the grayscale difference between segmented meibomian glands and the surrounding tarsal background; a lower value indicates reduced gland-to-background contrast and a less clearly visualized gland pattern (14). This parameter may capture imaging changes that precede overt gland dropout, but it is not yet a universally standardized clinical index and may be influenced by the optical characteristics of the surrounding tarsal tissue. Meibomian gland visualization and quantitative measurements may also vary with imaging modality. Edgar et al. compared anterior segment optical coherence tomography (AS-OCT) meibography with Keratograph 5M meibography and found strong test-retest reliability within each modality, but only fair agreement between subjective grading across modalities and relatively wide limits of agreement for quantitative gland-loss measurements, highlighting that imaging technology and acquisition characteristics can influence gland appearance and image-derived quantitative metrics (15). Furthermore, previous studies have mainly focused on changes in meibomian gland function or ocular surface parameters in TED, whereas few have investigated whether morphological changes in the levator palpebrae superioris complex (LPSC), the key anatomical structure involved in upper eyelid retraction, are quantitatively associated with meibomian gland morphology and tear parameters.

Upper eyelid retraction is a major clinical feature of TED and is present in more than 90% of affected patients (16, 17). Upper eyelid retraction can increase ocular surface exposure and alter eyelid dynamics, thereby affecting meibomian gland structure and lipid secretion (18, 19). The main pathological mechanisms underlying upper eyelid retraction include inflammation, thickening, and fibrosis of the LPSC (20). Therefore, morphological abnormalities of the LPSC may be associated with the structural status of the meibomian glands and ocular surface conditions. Identifying quantifiable relationships between these parameters may help improve our understanding of disease mechanisms.

In recent years, artificial intelligence (AI) has made substantial progress in ophthalmic image analysis (21–28) Deep learning-based meibomian gland segmentation models, such as ResNet50_U-Net, has been developed and validated in previous studies (14, 29, 30). This model can achieve 92% segmentation accuracy, assessed by intersection over union (IoU), and 100% repeatability for meibomian gland segmentation, and can automatically quantify morphological parameters such as meibomian gland density, height, and tortuosity. A multicenter study by Wang et al. showed that an attention-restricted neural network (ALNN)-based algorithm for tear meniscus height (TMH) measurement was highly correlated with the gold standard (*r* = 0.935–0.957) and achieved higher measurement accuracy than experienced clinicians (31). High-precision measurement of meibomian gland morphology and TMH may therefore serve as a quantitative method for evaluating ocular surface disease (32–34) These previously developed and validated models were applied as standardized quantitative measurement tools.

In the present study, we aimed to investigate the associations between ocular surface features, including meibomian gland morphology and TMH, and LPSC morphological parameters. We hypothesized that LPSC thickening in patients with TED and upper eyelid retraction may be associated with ocular surface parameters, including meibomian gland vagueness and density, and TMH, and that these relationships can be quantified by comparing AI-derived ocular surface morphology with CT-derived LPSC morphology.

## Materials and methods

### Study design

This was a single-center retrospective cross-sectional study. Patients diagnosed with TED-related upper eyelid retraction who underwent dry eye-related examinations and orbital CT at the Hangzhou Branch of the Eye Hospital, Wenzhou Medical University, between May 2023 and October 2024 were included. The study adhered to the Declaration of Helsinki and was approved by the Ethics Committee of the Eye Hospital, Wenzhou Medical University (approval No. H2026-021-K-21). The study was registered with the Chinese Clinical Trial Registry (registration No. ChiCTR2600123246). The requirement for informed consent was waived because of the retrospective design. This study was supported by the Medical and Health Science Program of Zhejiang Province (2025HY0596).

### Study population

The inclusion criteria were as follows: (1) diagnosis of thyroid-associated ophthalmopathy according to the Bartley criteria; (2) age ≥18 years and <70 years; and (3) an upper eyelid margin located at or above the superior corneal limbus in primary gaze, or a difference in upper lid marginal reflex distance (MRD1) of ≥1 mm between the affected eye and the contralateral eye.

The exclusion criteria were as follows: (1) dry eye caused by other conditions, such as Sjögren syndrome; (2) use of medications affecting the tear film or meibomian gland function within the preceding 3 months; (3) restrictive strabismus or clinically ocular movement restriction;(4) systemic or topical use of glucocorticoids, immunosuppressants, or biologic agents within the preceding 3 months; (5) contact lens wear; (6) periocular botulinum toxin injection within the preceding 6 months; and (7) meibomian gland, ocular surface, or CT images of insufficient quality for analysis.

### AI-based measurement and analysis of meibomian gland morphology

A convolutional neural network-based ResNet50_U-Net model was used. In this model, the encoder of the conventional U-Net architecture is replaced with a 50-layer residual network (ResNet50) to address the gradient vanishing problem in deep networks and to improve segmentation accuracy and feature extraction. Upper eyelid meibography was performed using the Keratograph 5M (Oculus, Wetzlar, Germany). Images were displayed in grayscale, stored in PNG format, and imported into the AI model for analysis (14, 29). The AI system automatically calculated meibomian gland vagueness and density. Meibomian gland vagueness was calculated by measuring the grayscale value of each pixel in the meibography image and determining the mean grayscale values of the meibomian glands and background tissue; vagueness was defined as the mean grayscale value of the meibomian glands minus the mean grayscale value of the background. A higher value indicates greater gland-to-background contrast and clearer gland visualization. The model did not include a separate normalization procedure specifically designed to correct grayscale values for regional tarsal or eyelid edema. Representative workflows for both AI analyses are summarized in Figure 1.

**Figure 1:**
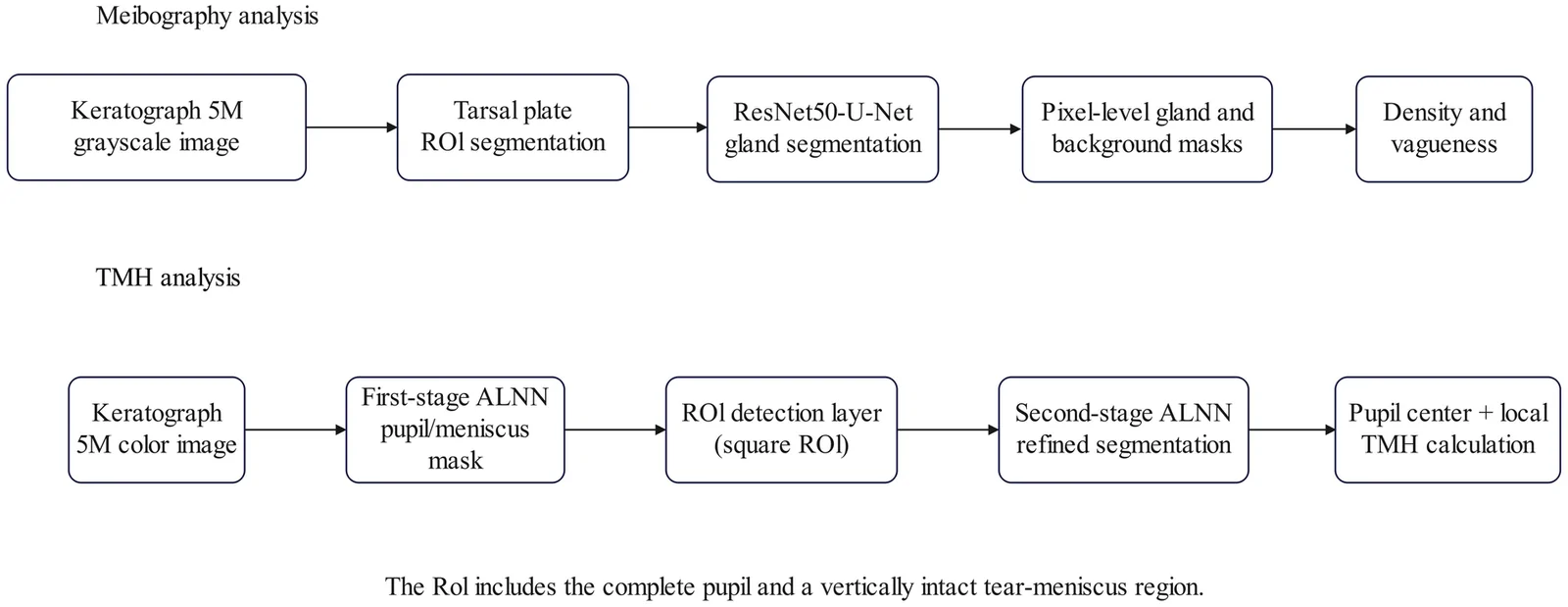
Schematic workflow of the AI-based meibomian gland and tear meniscus height analyses. ALNN, attention-limiting neural network; ROI, region of interest; TMH, tear meniscus height.

Calculation of meibomian gland vagueness and density (14):

- Mean grayscale value of meibomian glands=Total grayscale values of meibomian gland/Total meibomian gland pixels;
- Mean grayscale value of background=(Total grayscale values of tarsus - Total grayscale values of meibomian gland)/(Total tarsal pixels - Total meibomian gland pixels);
- Meibomian gland vagueness=Mean grayscale value of meibomian glands - Mean grayscale value of background.
- Meibomian gland density=Sum pixels of all Meibomian glands/Total pixels of the tarsus.

### AI-based measurement and analysis of TMH

An ALNN-based automated method was used to measure TMH. The model uses a two-stage architecture in which an ROI detection layer identifies a square region containing the complete pupil and the vertically intact tear meniscus, after which the ROI is processed for segmentation (31). This attention-limiting strategy enlarges the effective representation of the small target and reduces interference from irrelevant image regions. This model was previously developed and externally validated in a multicenter study including 3,894 images from five centers. For color images, the model achieved a mean intersection over union (MIoU) of 0.9578, recall of 0.9648, precision of 0.9526, and an F1 score of 0.9576 (31). Ocular surface images were acquired using the Keratograph 5M (Oculus, Wetzlar, Germany). All color images were acquired at a single center using the same Keratograph 5M system, processed through the same AI pipeline. Images were displayed at a resolution of 1,024 × 1,360 pixels. The pupil center was localized, and TMH was defined as the mean vertical distance between the upper and lower boundaries of the segmented tear meniscus within a narrow measurement section directly below the pupil center (Figure 1). This localized measurement reduces dependence on the contour of the entire lower tear meniscus; nevertheless, only images of sufficient quality for reliable pupil and tear-meniscus delineation were included.

### Orbital CT measurement and analysis

After contraindications were excluded and metallic objects from the head and face were removed, a Siemens 128-slice spiral CT scanner was used to continuously scan the craniofacial region from 10 mm above the superior orbital rim to 5 mm below the inferior orbital rim, ensuring complete coverage of the bony orbit and intraorbital soft tissues. The slice thickness was 0.625 mm. Coronal and sagittal multiplanar reconstructions were generated from the original data. Soft-tissue window images were selected at two coronal planes: the plane at the thickest portion of the LPSC near the globe equator, and the plane 6 mm posterior to the posterior pole of the globe. Images were exported to Image J software. After scale standardization, the LPSC contour was manually delineated because no validated automated or semi-automated LPSC segmentation software developed by our group was available for these CT images. The same investigator performed all measurements using the same anatomical definitions and was masked to the ocular surface analysis results. Each parameter was measured three times, and the mean value was used for analysis. Intra-observer repeatability was assessed using a two-way mixed-effects, absolute-agreement intraclass correlation coefficient (ICC). Inter-observer reproducibility was not assessed.

### Outcome measures

The primary outcome measures were meibomian gland vagueness and density, TMH, anterior LPSC thickness and area, and middle LPSC thickness and area. Secondary outcome measures included non-invasive tear break-up time (NBUT), which was automatically generated by the Keratograph 5M and included first NBUT and average NBUT. Covariates included CAS, proptosis, and MRD1.

### Statistical analysis

Normality was assessed using the Shapiro–Wilk test and histograms. Normally distributed variables are presented as mean ± standard deviation (M ± SD), whereas non-normally distributed variables are presented as median and interquartile range [M (Q1, Q3)]. Categorical variables are presented as counts and percentages. Pearson or Spearman correlation analyses and partial correlation analyses were used to explore the associations between LPSC parameters and meibomian gland morphological parameters, TMH, and NBUT. The results were visualized using heatmaps and scatterplots. Generalized estimating equations (GEE) were used to further evaluate the associations, with the participant as the subject variable and both eyes as repeated measures within subjects, while controlling for confounding factors. For selected variables, exploratory parallel mediation analyses were performed using the PROCESS macro (Model 4; Hayes, 2022). Bootstrap sampling was repeated 5,000 times to calculate 95% confidence intervals (CIs) for indirect effects. An indirect effect was considered significant if the 95% CI did not include 0. All analyses were conducted using SPSS version 26.0 and R version 4.5.2. Two-sided *P* values <0.05 were considered statistically significant.

## Results

### Demographic and clinical characteristics

A total of 67 patients (89 eyes) were included in this study. Among them, 22 patients contributed both eyes, and 45 patients contributed one eye (29 right eyes and 16 left eyes). There were 6 male and 61 female patients, and the mean age was 32.82 ± 8.12 years. In the study population, meibomian gland vagueness was 18.77 ± 4.61, meibomian gland density was 0.288 ± 0.06, and TMH was 0.26 (0.21, 0.32) mm. First NBUT was 5.35 (3.25, 8.55) s, and average NBUT was 9.84 ± 3.32 s. The anterior LPSC thickness and area were 3.65 ± 1.32 mm and 31.10 ± 13.91 mm^2^, respectively, while the middle LPSC thickness and area were 3.55 ± 1.11 mm and 28.62 ± 10.27 mm^2^, respectively (Table 1).

**Table 1:** Demographic and clinical characteristics of the study population (*n* = 67 patients, 89 eyes).

Variables	Mean ± SD/*n* (%)/Median (Q1, Q3)
Age (years)	32.82 ± 8.12
Sex (male/female)	6 (9.0%)/61 (91.0%)
Eye (OD/OS)	51 (57.3%)/38 (42.7%)
Graves’ disease/Hashimoto's thyroiditis	62 (92.5%)/5 (7.5%)
Duration of thyroid disease (months)	8 (3, 15.7)
Duration of upper lid retraction (months)	5 (2, 12)
Thyroid function (low/normal/high)	9 (13.4%)/31 (46.3%)/27 (40.3%)
History of smoking, *n* (%)	7 (10.4%)
CAS	1 (1, 2)
Proptosis (mm)	17.94 ± 2.60
MRD1 (mm)	5.53 ± 1.22
Meibomian gland vagueness	18.77 ± 4.61
Meibomian gland density	0.288 ± 0.06
TMH (mm)	0.26 (0.21, 0.32)
NBUT-first break (s)	5.35 (3.25, 8.55)
NBUT-average (s)	9.84 ± 3.32
Anterior thickness of LPSC (mm)	3.65 ± 1.32
Anterior area of LPSC (mm^2^)	31.10 ± 13.91
Middle thickness of LPSC (mm)	3.55 ± 1.11
Middle area of LPSC (mm^2^)	28.62 ± 10.27

CAS, clinical activity score; MRD1, upper lid marginal reflex distance; TMH, tear meniscus height; NBUT, non-invasive break-up time; LPSC, levator palpebrae superioris complex.

A history of smoking was documented in 7 participants (4 of 6 men and 3 of 61 women). Because smoking exposure was uncommon and highly imbalanced by sex, it was not entered as a separate covariate in the multivariable models.

The CT-derived LPSC measurements demonstrated excellent intra-observer repeatability. The ICCs were 0.962 (95% CI, 0.950-0.972) for anterior thickness, 0.970 (95% CI, 0.960–0.978) for anterior area, 0.971 (95% CI, 0.962–0.979) for middle thickness, and 0.978 (95% CI, 0.971–0.984) for middle area (all *P* < 0.001).

### Associations between ocular surface features and LPSC parameters

Heatmap analysis of the associations between ocular surface features and LPSC parameters showed that meibomian gland vagueness and TMH were associated with LPSC morphological parameters (Figure 2). The LPSC parameters associated with meibomian gland vagueness were anterior area (*r* = −0.333, *p* = 0.002), middle thickness (*r* = −0.287, *p* = 0.008), and middle area (*r* = −0.352, *p* = 0.001) (Figure 3). Meibomian gland density was not significantly associated with LPSC morphological parameters. The LPSC parameters associated with TMH were middle thickness (*r_s_* = 0.254, *p* = 0.017) and middle area (*r_s_* = 0.348, *p* < 0.001) (Figure 4). NBUT was not significantly associated with LPSC parameters. After adjustment for CAS, proptosis, and MRD1, partial correlation analysis showed that anterior LPSC area, middle LPSC thickness, and middle LPSC area remained negatively correlated with meibomian gland vagueness, whereas middle LPSC thickness and area were not significantly correlated with TMH (Table 2).

**Figure 2:**
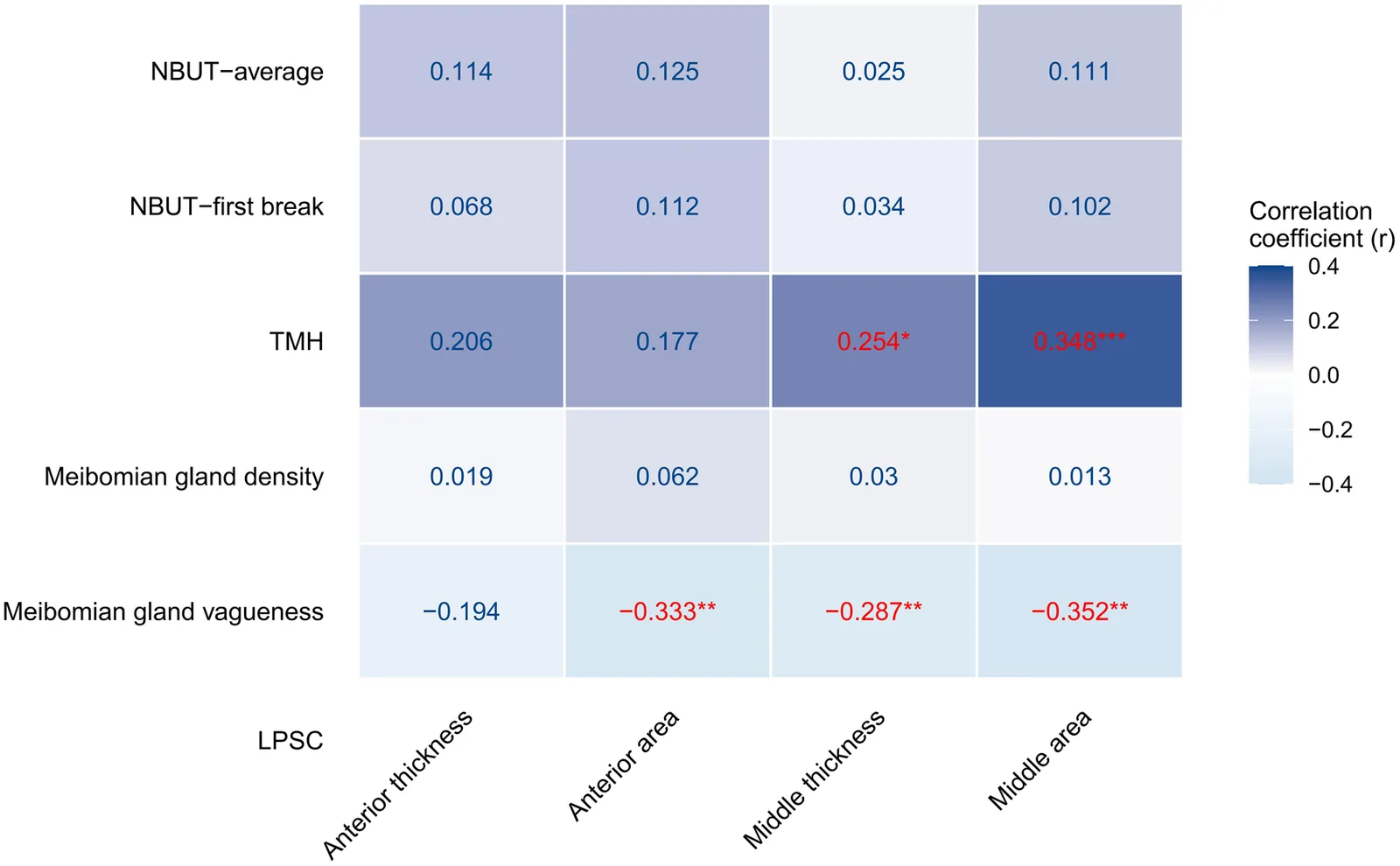
Heatmap of correlations between ocular surface features and LPSC morphological parameters. TMH, tear meniscus height; NBUT, non-invasive break-up time; LPSC, levator palpebrae superioris complex. *p < 0.05, **p < 0.01, ***p < 0.001.

**Figure 3:**
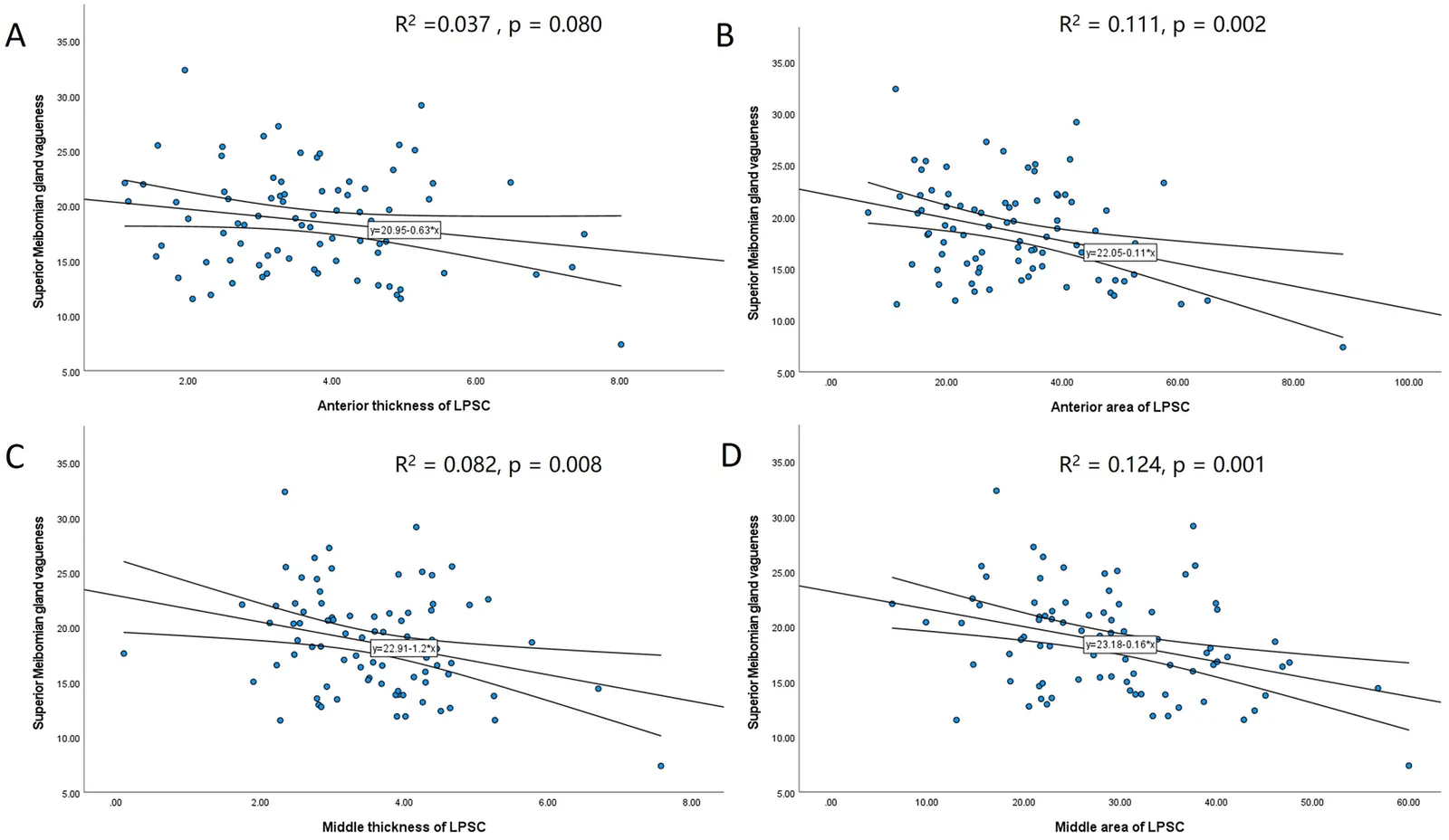
Scatterplots showing correlations between meibomian gland vagueness and LPSC morphological parameters. **(A)** anterior thickness, **(B)** anterior area, **(C)** middle thickness, **(D)** middle area. LPSC, levator palpebrae superioris complex.

**Figure 4:**
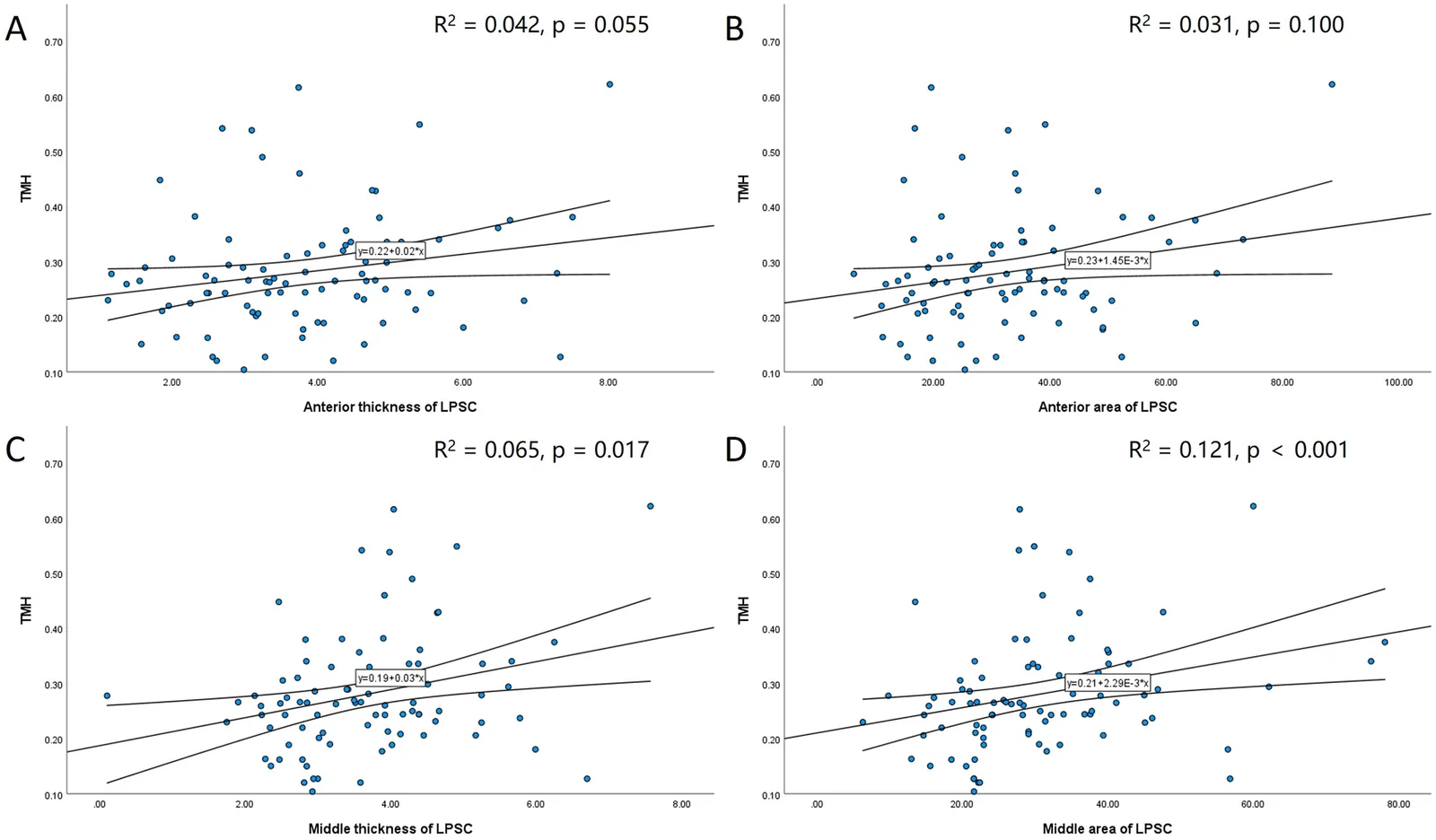
Scatterplots showing correlations between TMH and LPSC morphological parameters. **(A)** anterior thickness, **(B)** anterior area, **(C)** middle thickness, **(D)** middle area. TMH, tear meniscus height; LPSC, levator palpebrae superioris complex.

**Table 2:** Bivariate correlation analysis of meibomian gland, tear film, and LPSC parameters.

Variables	Anterior thickness *r*/*r_s_*, *p*	Anterior thickness *r*^a^/*r_s_*^a^, *p*^a^	Anterior area *r*/*r_s_*, *p*	Anterior area *r*^a^/*r_s_*^a^, *p*^a^	Middle thickness *r*/*r_s_*, *p*	Middle thickness *r*^a^/*r_s_*^a^, *p*^a^	Middle area *r*/*r_s_*, *p*	Middle area *r*^a^/*r_s_*^a^, *p*^a^
Meibomian gland vagueness	−0.194, 0.080	−0.210, 0.067	−0.333, **0.002**	−0.372, **<0.001**	−0.287, **0.008**	−0.297, **0.009**	−0.352, **0.001**	−0.369, **<0.001**
Meibomian gland density	0.019, 0.863	0.090, 0.437	0.062, 0.568	0.144, 0.211	0.030, 0.785	0.106, 0.361	0.013, 0.907	0.051, 0.660
TMH	0.206, 0.055	0.151, 0.190	0.177, 0.100	0.080, 0.492	0.254, **0.017**	0.193, 0.093	0.348, **<0.001**	0.201, 0.079
NBUT-first break	0.068, 0.533	0.000, 0.999	0.112, 0.307	0.060, 0.607	0.034, 0.759	−0.088, 0.447	0.102, 0.354	−0.016, 0.891
NBUT-average	0.114, 0.298	0.073, 0.529	0.125, 0.255	0.068, 0.556	0.025, 0.821	−0.071, 0.537	0.111, 0.312	0.054, 0.642

Bold values indicate statistical significance (*p* < 0.05).

a Partial correlation analysis adjusted for clinical activity score, upper lid marginal reflex distance, and proptosis. LPSC, levator palpebrae superioris complex; TMH, tear meniscus height; NBUT, non-invasive break-up time.

GEE analysis showed that the independent LPSC parameters associated with meibomian gland vagueness were anterior thickness (*β* = 1.504, 95% CI = 0.29 to 2.715, *p* = 0.015), anterior area (*β* = −0.168, 95% CI = −0.291 to −0.045, *p* = 0.008) and middle area (*β* = −0.144, 95% CI = −0.236 to −0.053, *p* = 0.002). LPSC parameters were not significantly associated with meibomian gland density or TMH (Tables 3, 4). Proptosis and MRD1 were independently positively and negatively associated with TMH, respectively (*β* = 0.016, 95% CI = 0.009 to 0.024, *p* < 0.001; and *β* = −0.025, 95% CI = −0.040 to −0.010, *p* = 0.001) (Table 5).

**Table 3:** Multivariate analysis of factors associated with meibomian gland vagueness.

Variable	β	95% CI lower	95% CI upper	*p*
Anterior thickness of LPSC	1.504	0.293	2.715	**0** **.** **015**
Anterior area of LPSC	−0.168	−0.291	−0.045	**0**.**008**
Middle thickness of LPSC	−0.356	−1.178	0.466	0.396
Middle area of LPSC	−0.144	−0.236	−0.053	**0**.**002**
CAS	0.244	−0.976	1.464	0.695
Proptosis	0.376	−0.008	0.759	0.055
MRD1	−0.193	−0.883	0.496	0.583

Bold values indicate statistical significance (*p* < 0.05).

GEE model adjusted for CAS, proptosis, and MRD1, with subject as the cluster variable and eye as the repeated measure. CI, confidence interval; LPSC, levator palpebrae superioris complex; CAS, clinical activity score; MRD1, upper lid marginal reflex distance; TMH, tear meniscus height; NBUT, non-invasive break-up time.

**Table 4:** Multivariate analysis of factors associated with meibomian gland density.

Variable	β	95% CI lower	95% CI upper	*p*
Anterior thickness of LPSC	−0.001	−0.019	0.017	0.907
Anterior area of LPSC	0.000	−0.002	0.002	0.664
Middle thickness of LPSC	0.001	−0.020	0.021	0.941
Middle area of LPSC	0.000	−0.003	0.002	0.676
CAS	−0.011	−0.034	0.012	0.353
Proptosis	0.005	−0.001	0.011	0.081
MRD1	−0.007	−0.020	0.005	0.258

GEE model adjusted for CAS, proptosis, and MRD1, with subject as the cluster variable and eye as the repeated measure. CI, confidence interval; LPSC, levator palpebrae superioris complex; CAS, clinical activity score; MRD1, upper lid marginal reflex distance.

**Table 5:** Multivariate analysis of factors associated with TMH.

Variable	β	95% CI lower	95% CI upper	*p*
Anterior thickness of LPSC	0.016	−0.006	0.038	0.160
Anterior area of LPSC	−0.001	−0.005	0.002	0.338
Middle thickness of LPSC	0.008	−0.013	0.029	0.469
Middle area of LPSC	0.001	−0.001	0.003	0.486
CAS	0.012	−0.013	0.037	0.354
Proptosis	0.016	0.009	0.024	**<0** **.** **001**
MRD1	−0.025	−0.040	−0.010	**0**.**001**

Bold values indicate statistical significance (*p* < 0.05).

GEE model adjusted for CAS, proptosis, and MRD1, with subject as the cluster variable and eye as the repeated measure. TMH, tear meniscus height; CI, confidence interval; LPSC, levator palpebrae superioris complex; CAS, clinical activity score; MRD1, upper lid marginal reflex distance.

### Mediation analysis

Based on the preceding analyses, middle LPSC thickness and area were positively associated with TMH in bivariate analyses. Meanwhile, GEE analysis showed that proptosis was positively associated with TMH, MRD1 was negatively associated with TMH, and middle LPSC thickness and area were not significantly associated with TMH. To further explore potential pathways linking middle LPSC thickness and area with TMH, exploratory mediation analyses were performed using middle LPSC thickness or area as the independent variable, TMH as the dependent variable, and proptosis and MRD1 as parallel mediators.

When middle LPSC thickness was used as the independent variable, the indirect effect through proptosis was significant (effect = 0.0092, 95% bootstrap CI: 0.0008 to 0.0203), accounting for 36.22% of the total effect. The indirect effect through MRD1 was not significant (effect = 0.0008, 95% CI: −0.0039 to 0.0066). The direct effect of middle LPSC thickness on TMH was not significant (effect = 0.0154, 95% CI: −0.0018 to 0.0326). These findings suggest that proptosis may participate in the statistical association between middle LPSC thickness and TMH (Table 6).

**Table 6:** Parallel mediation effect analysis of middle LPSC thickness on TMH.

Mediator	Path a (X→M)	Path b (M→Y)	Indirect effect	BootSE	95% Bootstrap CI lower	95% Bootstrap CI upper
Proptosis	0.5613*	0.0164***	0.0092*	0.0051	0.0008	0.0203
MRD1	−0.0346	−0.0234**	0.0008	0.0026	−0.0039	0.0066
Total indirect effect			0.0100	0.0058	−0.0002	0.0225
Direct effect			0.0154		−0.0018	0.0326

Bootstrap CI was based on 5,000 bootstrap samples. PROCESS Model 4. LPSC, levator palpebrae superioris complex; TMH, tear meniscus height; CI, confidence interval; MRD1, upper lid marginal reflex distance.

* *p* < 0.05.

** *p* < 0.01.

*** *p* < 0.001.

When middle LPSC area was used as the independent variable, the indirect effect through proptosis was significant (effect = 0.0008, 95% bootstrap CI: 0.0001 to 0.0019), accounting for 34.78% of the total effect. The indirect effect through MRD1 was not significant (effect = 0.0001, 95% CI: −0.0004 to 0.0021). The direct effect of middle LPSC area on TMH was not significant (effect = 0.0013, 95% CI: −0.0003 to 0.0029). These findings suggest that proptosis may participate in the statistical association between middle LPSC area and TMH (Table 7).

**Table 7:** Parallel mediation effect analysis of middle LPSC area on TMH.

Mediator	Path a (X→M)	Path b (M→Y)	Indirect effect	BootSE	95% Bootstrap CI lower	95% Bootstrap CI upper
Proptosis	0.0510*	0.0165***	0.0008*	0.0005	0.0001	0.0019
MRD1	−0.0064	−0.0229	0.0001	0.0003	−0.0004	0.0007
Total indirect effect			0.0010*		0.0001	0.0021
Direct effect			0.0013		−0.0003	0.0029

Bootstrap CI was based on 5,000 bootstrap samples. PROCESS Model 4. LPSC, levator palpebrae superioris complex; TMH, tear meniscus height; CI, confidence interval; MRD1, upper lid marginal reflex distance.

* *p* < 0.05.

*** *p* < 0.001.

## Discussion

This study demonstrated that upper eyelid meibomian gland vagueness in patients with TED was negatively correlated with anterior and middle LPSC area. After adjustment for proptosis, MRD1, and CAS, meibomian gland vagueness remained lower as anterior or middle LPSC area increased. Meibomian gland vagueness reflects the grayscale difference between the meibomian glands and surrounding tissue under infrared imaging and is closely related to the degree of edema in the surrounding tissue. The central pathological process of TED is autoimmune inflammation of affected tissues. Levels of pro-inflammatory cytokines, such as IL-1β, IL-6, IL-8, and TNF-α, are significantly increased in tears, and ocular surface inflammation may lead to edema of the tissue surrounding the meibomian glands and reduced infrared transmittance, thereby decreasing meibomian gland vagueness (1, 5, 35). LPSC thickening is caused by lymphocytic infiltration and extracellular glycosaminoglycan accumulation and may reflect local tissue inflammation (36). Because the LPSC and meibomian glands are located in the same upper eyelid anatomical region, local inflammation, tissue edema, or changes in the mechanical environment may jointly affect their imaging features. Previous studies have also shown that topical or systemic anti-inflammatory treatment can improve ocular surface inflammatory mediators, meibomian gland morphology, and CAS to some extent (37). Therefore, LPSC thickening and reduced meibomian gland vagueness may both represent manifestations of local inflammation in the upper eyelid.

However, the interpretation of meibomian gland vagueness is dependent on imaging technology and acquisition conditions. Different meibography modalities may differ in illumination, optical path, field of view, post-processing, and contrast characteristics, which can alter gland visualization and image-derived quantitative measurements. In Edgar's study, its was noted that K5M incorporated image processing illustrated how device-specific processing can affect gland appearance (15). Such factors are especially relevant to vagueness because this metric is calculated directly from the grayscale difference between gland and background tissue. In the present study, all meibography images were obtained at a single center using the same K5M system and analyzed with the same AI pipeline; nevertheless, image quality, focus, eyelid eversion, illumination, contrast characteristics, and other acquisition-related factors may still influence the measured vagueness value. Accordingly, vagueness values should be interpreted within the context of the specific imaging modality and acquisition protocol used, and their cross-device comparability requires further validation.

In contrast to meibomian gland vagueness, meibomian gland density was not associated with LPSC morphological parameters in this study. Meibomian gland density primarily reflects structural damage and atrophy of the glands, whereas vagueness reflects gland-to-background image contrast and may change before measurable dropout. In the AI validation study by Zhang et al., meibomian gland density showed an area under the curve of 0.900 for identifying meibomian gland dysfunction, with a proposed cutoff of 0.275. The mean density in the present cohort was 0.288 ± 0.06, and the median duration of upper eyelid retraction was only 5 months (12, 18, 29). Yin and Gong reported that meibomian gland vagueness was significantly lower in patients with meibomian gland dysfunction than in matched healthy controls and was correlated with several clinical and morphological indices (38). In the present study, lower vagueness was associated with larger LPSC areas even after adjustment for CAS, proptosis, and MRD1. Together with previous evidence that greater gland loss occurs in moderate-to-severe or longer-standing TED, one possible interpretation is that reduced gland-background contrast represents an early or inflammation-sensitive imaging change that precedes measurable gland loss. Therefore, LPSC enlargement may be more closely associated with meibomian gland vagueness than with density in this relatively early, eyelid-predominant cohort. This interpretation remains hypothesis-generating because the cross-sectional design cannot determine the temporal sequence of these imaging changes.

Bivariate analyses showed that TMH was positively correlated with middle LPSC thickness and area. However, after adjustment for proptosis, MRD1, and CAS, partial correlation and GEE analyses did not show a direct association between these variables. Mediation analysis suggested that proptosis may participate in the statistical association between middle LPSC thickness or area and TMH, with indirect effects accounting for 36.22% and 34.78% of the total effects, respectively. Proptosis reflects an increase in retrobulbar relative volume and is closely related to the volumes of extraocular muscles and orbital fat. Ismailova et al. reported that increased proptosis may exacerbate ocular surface exposure, thereby enhancing stimulation of corneal sensory nerve endings and inducing compensatory reflex tear secretion (1). Although patients with exposure keratitis were excluded from the present cohort, subclinical ocular surface exposure associated with proptosis or upper eyelid retraction may still have contributed to reflex tearing. Though GEE analysis suggested that MRD1 may also be involved in TMH changes, mediation analysis did not identify a significant indirect effect through MRD1. This may indicate that the contribution of MRD1 to tear secretion is smaller than that of proptosis and may therefore be diluted in the mediation model.

No significant association was found between NBUT and LPSC morphological parameters. NBUT reflects tear film stability and is influenced by the lipid, aqueous, and mucin layers. It is not only related to excessive evaporation associated with upper eyelid retraction and meibomian gland dysfunction but also affected by reduced ocular surface mucin, corneal nerve injury, and incomplete blinking (7, 11, 39–41) Therefore, NBUT may be more appropriate as a composite functional indicator than as a direct reflection of changes in a single anatomical structure.

The low CAS distribution in this cohort was largely a consequence of the predefined phenotype rather than an attempt to represent the full clinical spectrum of TED. Patients with restrictive strabismus, dysthyroid optic neuropathy, exposure keratopathy, so that the analysis would primarily reflect isolated upper eyelid retraction and reduce interference from other orbital manifestations. Accordingly, the observed associations are most applicable to patients with mild or inactive disease and should not be extrapolated to clinically active, severe, or multi-phenotype TED.

Blink mechanics may constitute an important mechanical pathway linking LPSC abnormalities to meibomian gland changes. Effective blinking distributes the tear-film lipid layer and provides repeated compression of the tarsal plate, facilitating meibum delivery from the gland ducts. Upper eyelid retraction, increased palpebral fissure height, and LPSC inflammation or fibrosis may reduce blink completeness and alter lid-globe apposition. Previous work in TED has linked increased incomplete blinking with meibomian gland loss (11). Thus, impaired blink-related gland expression may act together with local inflammation to reduce gland clarity and destabilize the tear film. Because blink frequency and incomplete blink rate were not recorded in this study, this proposed mechanical pathway could not be formally evaluated.

This study has several limitations. First, it was a single-center retrospective study, and causal relationships between LPSC morphology and ocular surface parameters cannot be inferred. Second, the limited sample size and predominantly mild or inactive TED cases restricted the ability to include additional covariates in multivariable analyses and to perform more detailed subgroup analyses, such as active versus inactive disease or mild versus moderate-to-severe disease. The cohort was also markedly female-predominant (61 of 67 participants), which may limit generalizability and precluded robust sex-stratified analyses. Third, only two coronal CT planes were selected to analyze LPSC thickness and area, which may limit the representation of true muscle morphology. Fourth, the meibomian gland vagueness algorithm did not specifically normalize grayscale values for regional tarsal or eyelid edema. Fifth, TMH images were acquired without topical anesthesia, and the retrospective protocol did not distinguish basal tear volume from reflex tear secretion. Sixth, the ALNN measures a localized tear-meniscus segment directly below the pupil center, which was not specifically validated in TED eyes with markedly discontinuous or distorted tear menisci. The source model also acknowledged reduced robustness for abnormal meniscus shapes and very high TMH values; therefore, residual segmentation error may remain despite exclusion of images judged insufficient for analysis. Seventh, tear samples were not collected for inflammatory cytokine testing, and serological markers such as TRAb and TSI, which are important markers of TED activity and may be related to meibomian gland morphology and function, were not included; consequently, the molecular link between LPSC inflammation and ocular surface imaging changes could not be established. Finally, blink dynamics, including incomplete blink rate and blink frequency, were not measured, preventing quantitative evaluation of the mediating role of blinking mechanisms between LPSC thickening and meibomian gland changes.

## Conclusion

In patients with TED and upper eyelid retraction, LPSC thickening was associated with reduced meibomian gland vagueness. The association between LPSC thickening and increased TMH was not direct, and proptosis may participate in the relationship between these parameters. However, these findings require further validation in multicenter, large-sample, longitudinal studies.

## Data Availability

The raw data supporting the conclusions of this article will be made available by the authors, without undue reservation.
